# Triple perspective: assessing deep brain stimulation outcomes in Parkinson's disease

**DOI:** 10.1186/s12883-025-04455-3

**Published:** 2025-10-14

**Authors:** Denise Mellace, Francesca Mameli, Fabiana Ruggiero, Filippo Cogiamanian, Linda Borellini, Angelica De Sandi, Angelica Marfoli, Eleonora Zirone, Enrico Mailland, Elena Pirola, Antonella M. Ampollini, Luigi G. Remore, Marco Locatelli, Alberto Priori, Sergio Barbieri, Roberta Ferrucci

**Affiliations:** 1 Department of Oncology and Hemato-Oncology, University of Milan, Via Santa Sofia 9/1, Milan, 20122 Italy; 2 Department of Neurosciences and Mental Health, Foundation IRCCS Ca’ Granda Ospedale Maggiore Policlinico, Via Francesco Sforza 35, Milan, 20122 Italy; 3 Department of Pathophysiology and Transplantation, University of Milan, Via Francesco Sforza 35, Milan, 20122 Italy; 4 SC Neurology I, ASST - Santi Paolo e Carlo, Via Antonio di Rudinì 8, Milan, 20142 Italy; 5 Department of Health Sciences, “Aldo Ravelli” Center for Neurotechnology and Brain Therapeutics, University of Milan, Via Antonio di Rudinì 8, Milan, 20142 Italy

**Keywords:** Parkinson’s disease, Deep brain stimulation, Treatment outcome, Patient outcome assessment, Visual analog scale

## Abstract

**Background:**

Deep brain stimulation (DBS) of the subthalamic nucleus (STN) is an established treatment for advanced Parkinson’s disease (PD), often leading to positive motor and non-motor outcomes. While objective motor improvements after DBS are well documented, less is known about how patients and all those involved in their care perceive these benefits on a subjective level.

**Objectives:**

The primary aim of the study was to investigate the perception gap between patients, caregivers, and treating neurologists regarding DBS physical and psychological benefits in PD and their correlates.

**Methods:**

25 PD patients (age 58.9 ± 8.0 years; 9 women) who underwent bilateral STN-DBS, along with their caregivers and neurologists, rated perceived psychological and physical improvements 6 months after surgery using a two-item Visual Analogue Scales (VAS, 0–10). Intraclass correlation coefficients (ICC [95% confidence interval]) were calculated to assess reliability between raters.

**Results:**

Patients, caregivers, and neurologists reported an average improvement of about 60% in the psychological domain and over 75% in the physical domain (*p* *< 0.001*). No significant differences emerged between groups in two domains (*p > 0.05*). Inter-rater agreement was moderate-to-good for psychological improvement (0.74 [0.41–0.90], *p* < 0.001), moderate for physical improvement (0.69 [0.27–0.88], *p* = 0.003), and good overall (0.79 [0.50–0.92], *p* < 0.001).

**Conclusions:**

PD patients, caregivers, and neurologists largely agree on the benefits of DBS six months post-surgery, reinforcing the reliability of patient self-report in outcome assessment. Integrating patient self-reports with proxy assessments enhances the evaluation of DBS outcomes, supporting a more comprehensive and patient-centered approach to both treatment assessment and post-surgical care.

**Trial registration:**

ClinicalTrials.gov Identifier: NCT06329726. Registered on 26 March 2024.

**Supplementary Information:**

The online version contains supplementary material available at 10.1186/s12883-025-04455-3.

## Introduction

Parkinson’s disease (PD) is a progressive neurodegenerative disorder primarily managed with pharmacological treatment. However, after approximately 5 years, many patients experience drug-related motor complications, limiting the effectiveness of pharmacological approaches [1]. Advanced stages of PD are characterized by increased motor and non-motor symptom burden, longer “off” time (periods when medication effects wear off and symptoms re-emerge), dyskinesia, greater disability in activity of daily living, and reduced quality of life [2]. In this context, deep brain stimulation (DBS) of the subthalamic nucleus (STN) has become a well-established intervention, effectively alleviating motor symptoms and reducing medication requirements. Clinical studies have shown that DBS reduces “off” time by 4–6 h per day, decreases motor fluctuations and dyskinesias by about 60%, and provides benefits on non-motor symptoms [1] such as mood, pain, and sleep, and overall quality of life [3, 4].

Despite its well documented clinical benefits, DBS can lead to challenges beyond motor improvement. Patients often face difficulties in personal and socio-professional adjustment [5], and motor symptom relief does not always translate into a positive subjective outcome [6]. Since DBS is an elective rather than an urgent medical procedure, the decision to undergo surgery is significant and requires careful consideration of potential benefits, risks, and impact on quality of life. Capturing patients’ experiences is essential for a comprehensive treatment assessment and for delivering patient-centered care [7].

Individual perceptions of DBS outcomes are inherently subjective and vary widely, as they are influenced by disease characteristics, personal circumstances, and perceived health status [8]. While many patients report satisfaction with short-term outcomes, particularly in terms of motor symptoms and quality of life [6, 9, 10], subjective evaluation may differ from patient to patient. To gain a more holistic understanding of DBS impact, it is essential to integrate multiple perspectives, including those of caregivers and neurologists who are closely involved in the perioperative and post-surgical care of patients [11]. Caregivers and clinicians provide valuable proxy reports, especially in cases where cognitive or communication impairments may limit the accuracy of self-reports [12]. Their evaluations complement patient-reported outcomes, offering a broader understanding of treatment effects and contributing to a multidimensional approach to DBS outcome assessment. Evaluating DBS outcomes from these perspectives could provide a valuable clinical reference point, yet research on the agreement between PD patients, caregivers, and neurologists regarding perceived STN-DBS treatment outcomes remains limited.

This study aims to address this gap by investigating the perception gap between patients, caregivers, and treating neurologists regarding DBS physical and psychological outcomes in PD six months post-DBS and their associations with changes in quality of life, mood, and other patient characteristics. By examining these perception gaps and their relationship with mood and cognitive factors, this study provides insights that may help tailor patient-centered care.

## Methods

Consecutive patients with advanced PD who underwent bilateral STN-DBS with continuous stimulation between 2017 and 2020 were enrolled in this study. Patients were prospectively assessed before STN-DBS and subsequently at 6 months follow-up. In addition, for each patient, their caregiver and neurologist experienced in movement disorders and invasive brain stimulation participated in the study. Patients were included based on Core Assessment Program for Surgical Interventional Therapies in PD (CAPSIT-PD) criteria, aged over 18, of either sex, with no hardware removal (explantation) due to complications, and who completed follow-up visits at our center. All procedures were carried out in accordance with the principles of the Declaration of Helsinki. The research was approved by the local Institutional ethics committee (Approval No. 2571, 16 November 2021) and informed consent was obtained from all participants. This study was registered at ClinicalTrials.gov (Identifier: NCT06329726) on 26 March 2024.

### Procedure

All patients underwent a preoperative (T0) comprehensive diagnostic screening according to the CAPSIT-PD [13] by a multidisciplinary team. A motor, cognitive, and psychodiagnostics assessment was performed to test dopaminergic responsiveness, rule out the presence of cognitive deficits, and minimize the risk of psychiatric complications.

Bilateral STN-DBS surgery was performed by a dedicated neurosurgical team following a standardized two-stage protocol [14]. In the first stage, leads were implanted under local anesthesia in awake patients. STN targeting was based on preoperative MRI (T1, T2, and FLAIR sequences) fused with stereotactic CT images using a dedicated planning workstation (Brainlab). Microelectrode recordings (FHC Inc.) were used for intraoperative mapping, and final electrodes (Medtronic 3389) were positioned accordingly. Position was tested by recording neurophysiological activity. In the second stage, performed under general anesthesia, the implantable pulse generator and extensions were placed. Stimulation was activated 3–6 weeks postoperatively during a second hospitalization.

The neuropsychological battery was partially repeated six months after electrode implantation (T1), specifically focusing on the Montreal Cognitive Assessment (MoCA) [15] to evaluate cognitive functioning, the Beck Depression Inventory II (BDI-II) [16] to assess mood, and the Parkinson’s Disease Questionnaire-8 (PDQ-8) [17] to measure quality of life. The PDQ-8 Summary Index (SI) was calculated as (total score/32) × 100, with higher scores indicating worse quality of life. During follow-up at T1, patients were asked to rate the perceived degree of improvement using a visual analogue scale (VAS) with two items, one related to the psychological domain and one related to the physical domain. The VAS was paper-based, presented as a horizontal line, and scores ranged from 0 (no improvement) to 10 (major improvement). The respondents indicated their perceived state by placing a mark at the most appropriate point along the line [18]. This two-item VAS was developed ad hoc to enable a simple and comparable evaluation across respondents. Visual analogue scales are commonly used in clinical settings to assess perceived changes over time and are considered reliable for capturing subjective impressions [18, 19]. At the same time, after the neurological and neuropsychological examination, the caregiver, and the neurologist – both blind to the patients’ responses and ratings – were asked to rate the patient’s improvement from their perspective on the identical VAS instrument. Patients, their caregivers, and neurologists independently assessed subjective perceptions of improvement in terms of psychological/cognitive benefits and physical/motor changes.

**Fig. 1:**
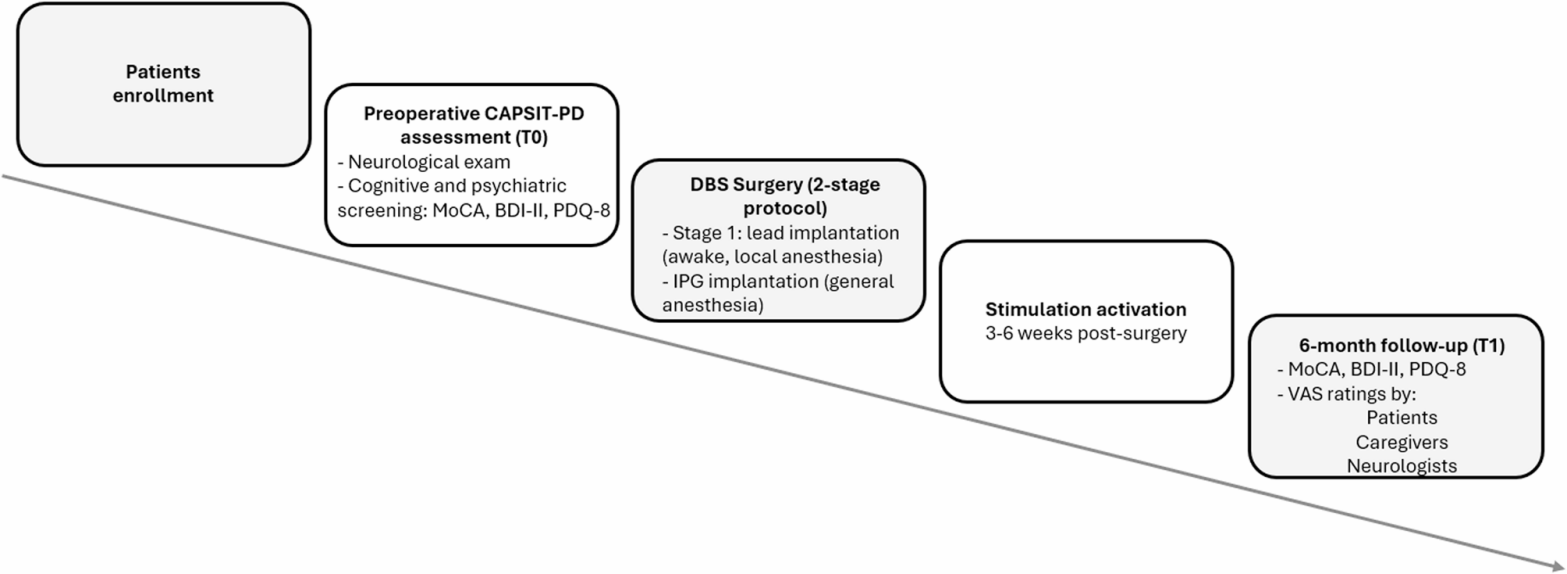
Overview of the study procedure. Notes. CAPSIT-PD = Core Assessment Program for Surgical Interventional Therapies in PD; MoCA = Montreal Cognitive Assessment; BDI-II = Beck Depression Inventory II; PDQ-8 = 8-item Parkinson’s Disease Questionnaire; IPG = Implantable Pulse Generator; VAS = Visual Analogue Scale

A schematic overview of the study procedure, including preoperative screening, surgical steps, and post-operative assessments, is provided in Fig. 1.

### Statistical analysis

Raw variables were checked for Normality by addressing skewness and kurtosis values, which were judged as abnormal if >|1| and |3|, respectively [20]. Due to non-normally distributed variables and to the limited sample size, non-parametric approaches were used to analyze significant differences between baseline versus 6-months follow-up (i.e., Wilcoxon test); to compare patients’, caregivers’, and neurologists’ scores on the psychological and physical VASs (i.e., Friedman’s test); and to test their association with demographic (i.e., sex, age, education) and clinical characteristics (i.e., disease duration in years, MoCA, BDI-II, and PDQ-8) (i.e., Spearman’s correlation).

The agreement rate between patients’, caregivers’ and neurologists’ scores was then tested *using* intra-class correlation coefficients (ICC). The ICC estimates the reliability of ratings between raters—the higher the correlation, the greater the agreement. Values below 0.5 indicate poor reliability, values between 0.5 and 0.75 indicate moderate reliability, values between 0.75 and 0.9 indicate good reliability, and values above 0.90 indicate excellent reliability [21].

Additionally, to control for potential confounding variables (age, sex, disease duration, cognitive and mood scores), two generalized linear models (GLMs) were performed with Gaussian distribution, using perceived physical and psychological improvement as dependent variables, and age, disease duration, cognition, mood, and quality of life as covariates.

Analyses were performed using jamovi 2.3 (the jamovi project, 2022) and IBM SPSS Statistics 29 (IBM Corp., 2023).

## Results

A total of 25 patients were included in this study. Table 1 summarizes the participants’ demographic and clinical data. Detailed demographic and clinical information for each patient is provided in Additional file 1. Comparison between baseline and 6-month follow-up showed no difference in the mean MoCA total score (*p* = 0.940). However, a significant 24% reduction was observed in the BDI-II total score (*p* = 0.019), as well as a significant 32% decrease in the PDQ-8 total score (*p* < 0.001) at T1.

**Table 1:** Patient’s demographic characteristics and clinical data

Variable	Mean ± SD (range)	*p*-value
N	25	
Age (years)	58.9 ± 8.03 (46–71)	
Sex		
Male	64%	
Female	36%	
Disease Duration	11.6 ± 3.55 (5–19)	
MoCA		
Pre	26 ± 2.42 (21–30)	0.940
Post	26.1 ± 2.78 (21–30)	
BDI-II		
Pre	9.24 ± 5.70 (2–20)	0.019*
Post	6.71 ± 4.89 (1–21)	
PDQ-8		
Pre	36.5 ± 16.2 (6.25–71.9)	< 0.001
Post	22.6 ± 15.2 (0-56.3)	

*MoCA *Montreal Cognitive Assessment, *BDI-II* Beck Depression Inventory II, *PDQ-8* 8-item Parkinson’s Disease Questionnaire

* *p* < 0.05

### How patients, caregivers, and neurologists perceive DBS outcomes

A total of 25 patients responded to the two-item VAS six months after DBS. Specifically, they reported a mean psychological improvement score of 6.4 ± 3.0, and a mean physical improvement score of 7.7 ± 1.9. The physical domain scores were significantly higher than the psychological domain scores (*p* *= 0.039*).

In addition, 21 caregivers answered the two-item VAS six months after DBS referring to the patient’s improvement. They rated psychological improvement with a mean score of 6.1 ± 2.6 and physical improvement with a mean score of 7.1 ± 2.1. The physical domain scores were significantly higher than the psychological domain scores (*p = 0.046*).

The three experienced neurologists who participated in the study provided answers to the two-item VAS for 20 patients. They rated the psychological domain with a mean score of 5.9 ± 2.8 and the physical domain with a mean score of 8.1 ± 1.3. The physical domain scores were significantly higher than the psychological domain scores (*p = 0.012*).

GLM analyses showed that most demographic and clinical variables were not significant predictors of perceived improvement. Only age was significantly associated with perceived physical improvement (χ²(1) = 4.39, *p* = 0.036), while no predictors were significant for perceived psychological improvement.

### Between-Group comparison and agreement rate

**Fig. 2:**
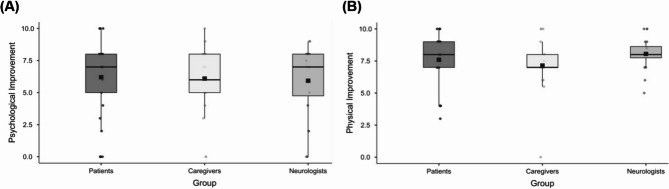
Boxplots illustrating the distribution of perceived improvement scores in (**A**) psychological and (**B**) physical domains 6 months after DBS among the three raters

When determining the agreement rate, only the subset of 20 patients with complete triads were considered. Missing data were primarily due to some patients attending unaccompanied, which prevented caregiver assessments, and occasional absence of a neurologist during follow-up visits. The concordance between the three raters ranked moderate-to-good for psychological improvement (ICC = 0.74; 95% CI [0.41, 0.90]; *p* < 0.001), moderate for physical improvement (ICC = 0.69; 95% CI [0.27, 0.88]; *p* = 0.003), and good for overall improvement (ICC = 0.79; 95% CI [0.50, 0.92]; *p* < 0.001) (Fig. 2).

Correlation analysis showed a strong and significant correlation between caregiver and neurologists in psychological scores (*r*_*s*_(14) = 0.71, *p* = 0.002). Additionally, a trend towards significant negative correlation between physical improvement score given by patients and BDI-II scores at T1 (*r*_*s*_(19)=−0.43, *p* = 0.0497) was found. No further significant correlations were found between subjective perceptions and demographic and clinical variables.

**Fig. 3:**
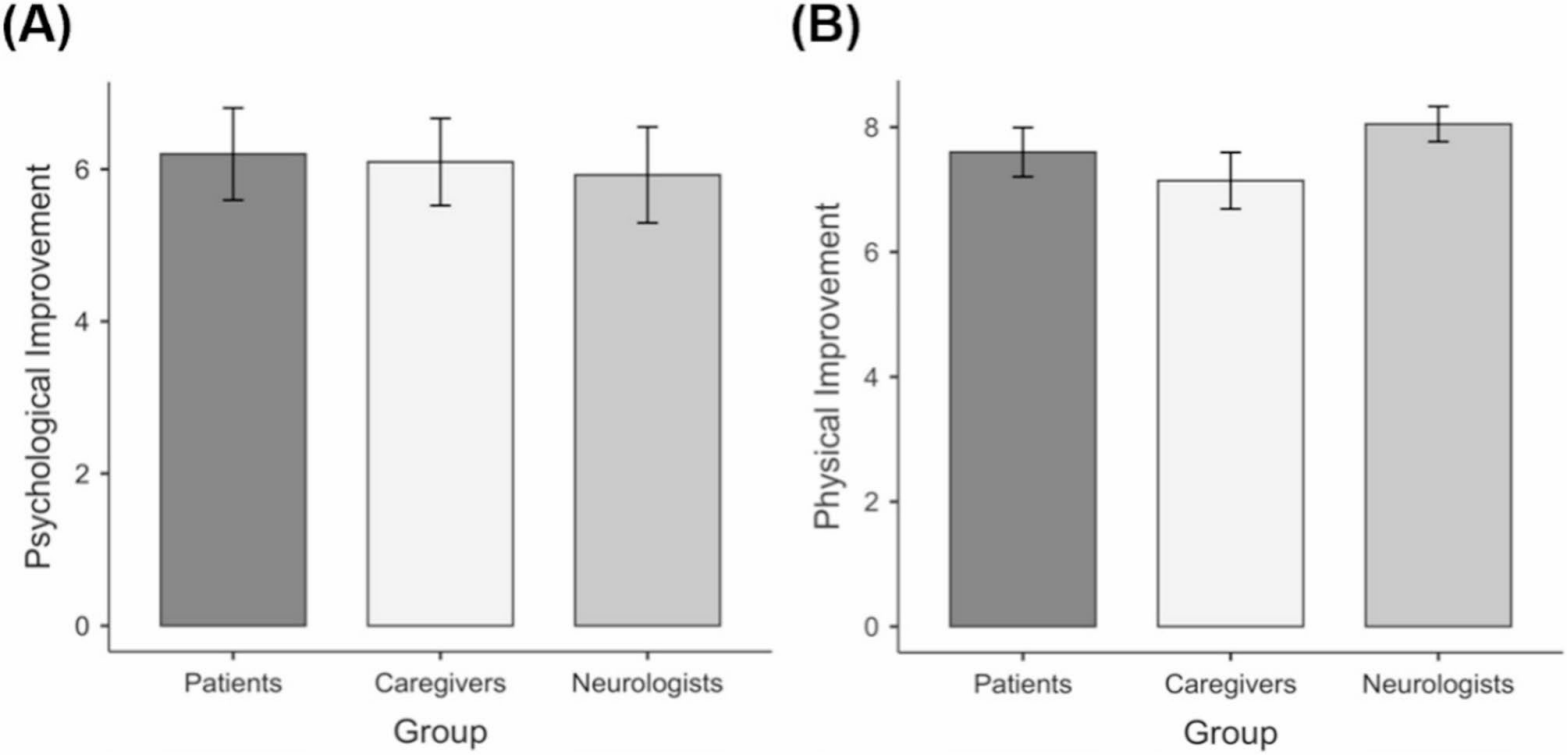
Psychological (**A**) and physical (**B**) improvement perceived by patients, caregivers, and neurologists. No significant differences between the scores of the three raters (psychological: *p* = 0.288; physical: *p* = 0.557).

Non-parametric repeated measures ANOVA showed no significant differences between raters in two domains (Fig. 3). Between-group comparisons were not significant for psychological (*χ*²(2) = 2.49; *p* = 0.288) or physical (*χ*²(2) = 1.17; *p* = 0.557) improvement.

## Discussion

This study examined the multidimensional outcomes of DBS in PD by integrating clinical measures with subjective perceptions of improvement from patients, caregivers, and neurologists six months post-surgery. The findings provide important insights into the benefits and limitations of DBS, particularly its psychological and physical impacts. Additionally, we evaluated the reliability of proxy assessments from caregivers and neurologists with patient self-reports by examining their agreement with patient self-reports.

Subjective ratings on VAS revealed that patients, caregivers, and neurologists consistently perceived a positive impact of DBS on PD patients six months after DBS, particularly in the physical domain, where improvements exceeded 75%. Ratings in the psychological domain were relatively lower (around 60%). These findings aligns with prior research showing stronger satisfaction with motor outcomes compared to psychological outcomes [22] in the short-term [9, 10]. Despite this, there is evidence indicating that motor gains do not always correlate with overall treatment satisfaction [23]. Notably, the relatively lower ratings in the psychological domain may reflect the persistence of non-motor symptoms such as mood and anxiety issues, which are less responsive to DBS [10]. Additionally, some patients in our sample without baseline cognitive or behavioral symptoms may have rated no improvement simply because that domain was not initially impaired. These nuances highlight that perceived improvement does not only reflect a measure of clinical efficacy but also depends on individual differences in the perception of change and personal expectations.

Reliability analysis showed significant agreement among patients, caregivers, and neurologists, with moderate-to-good agreement across all domains, indicating that patient’s self-assessments were largely consistent with proxy assessment from caregivers, who provide support and care and observe patients daily, and neurologists, who rely on clinical examination and scales [24]. These level of concordance suggests that patients appeared capable of accurately evaluating their improvement six months post-surgery, showing good insight into their condition without overestimating or underestimating the real benefit of DBS, likely supported by the absence of cognitive deficits or severe depressive symptoms, factors that could otherwise compromise self-assessment accuracy. The high level of agreement strengthens the argument that patients with no major neuropsychiatric comorbidities can serve as reliable informants of their own postoperative status [11, 25].

Our generalized linear models did not find significant associations between perceived psychological or physical improvements and most clinical or demographic variables, including disease duration, cognitive scores, and depression levels, except for a weak effect of age on physical outcome. These findings suggest that subjective perceptions of improvement after DBS are largely independent of these factors in our sample. However, the limited sample size and model constraints may have affected the power to detect smaller effects. Future studies with larger cohorts are needed to confirm these observations.

To our knowledge, this is the first study to investigate concordance between self-assessment, caregiver evaluation, and clinician judgement in the context of invasive neuromodulation. The inclusion of these three perspectives offers an enriched understanding on the accuracy and reliability of self-reported outcomes, which is particularly relevant when evaluating interventions aimed at improving quality of life.

A trend-level association was observed between patients perceived physical improvement and their post-DBS BDI-II scores. No correlations were found with demographic characteristics, disease duration, or cognitive efficiency. The negative nature of this relationship suggests that residual depressive symptoms may slightly affect how patients perceive their motor benefits, and mood improvement may positively influence motor benefits perception. While this observation aligns with previous findings which have shown that depression can negatively impact patients’ perception of progress, even when objective improvements are documented [26, 27], the borderline statistical significance calls for cautious interpretation and further investigation.

Interestingly, we found a strong correlation between caregivers’ and neurologists’ assessments of psychological outcomes, suggesting that these two groups share a similar perspective on patient’s emotional and mental well-being after DBS. This may reflect the tendency of both caregiver and clinicians to perceive patients’ psychological status after DBS treatment with a comparable level of (in)accuracy [28]. In contrast, the weaker correlations between these assessments and patient self-reports could indicate differences in self-perception versus external evaluation, particularly when it comes to emotional and psychological aspects. These findings show that proxies are generally more reliable in assessing observable symptoms, such as pain, but less accurate in evaluating subjective experiences, such as anxiety and depressive symptoms [25]. Therefore, although proxy reports are always valuable, direct patient involvement remains essential in assessing psychophysical well-being after DBS.

The results from standardized clinical scales, specifically the BDI-II and PDQ-8, showed a significant reduction in depressive symptoms and improvement in quality of life six months post-DBS. These findings are in line with previous studies reporting the short-term positive impact of DBS on mood and overall well-being in PD patients [10, 29, 30].

Finally, when discussing clinical outcomes, pre-intervention beliefs and expectations play a key role in shaping personal perceptions of improvement. Patients’ evaluation of their health is influenced not only by objective symptom changes and disease progression, but also by their expectations regarding treatment outcomes [31]. These expectations can affect satisfaction with DBS, particularly in specific functional areas. Hasegawa et al. [32] found a strong correlation between expectations and satisfaction in the short-term, whereas others did not confirm this in the long term [33]. Interestingly, motor improvements—which are the primary target of DBS—do not necessarily correlate with greater treatment satisfaction [10]. This highlights the complexity of patient perceptions, shaped by multiple factors beyond symptom relief, including psychological well-being and expectation fulfillment. Managing preoperative expectations is therefore crucial to optimize both satisfaction and clinical outcomes [22, 34]. Future research should further explore this relationship to refine patient counseling strategies.

In conclusion our findings indicate substantial concordance among the three groups, suggesting that patients’ self-reports are largely consistent with caregiver and neurologist assessments. This alignment reinforces the reliability of subjective evaluations in DBS outcome assessment and highlights the potential of integrating patient-reported outcomes into personalized treatment strategies and post-intervention rehabilitation protocols. While proxy assessments are often considered essential when self-reports may be compromised by cognitive or communication impairments, the observed agreement in our study suggests that, in cognitively intact patients, self-reports can serve as a reliable measure of perceived benefit.

This study has some limitations. The relatively small sample size may affect the generalizability of the findings. Additionally, the lack of standardized clinical rating scales for direct comparison with subjective reports, particularly in motor evaluations, limits interpretability. The absence of baseline subjective references also restricts longitudinal comparisons. Furthermore, the phrasing of items focused on perceived improvement, which may have unintentionally biased responses toward positive changes.

Future research should explore factors that influence subjective perceptions of DBS over time, including preoperative expectations and clinical characteristics, and examine whether agreement rates are affected by expectations or treatment satisfaction. A longitudinal approach could be valuable to track patients’ perceptions of DBS outcomes over an extended period and assess the validity of proxy evaluation in the long term. Additionally, comparing this cohort with PD patients who underwent DBS targeting alternative structures could provide further insight into how different stimulation sites influence subjective outcomes.

## Supplementary Information

Supplementary Material 1.

## Data Availability

Datasets related to the current study are available upon reasonable request from interested researchers.
