# Supplementary material for: Triple perspective: assessing deep brain stimulation outcomes in Parkinson's disease
Source: BMC Neurol. 2025 Oct 14;25:426. doi: 10.1186/s12883-025-04455-3 (PMC12522908; doi:10.1186/s12883-025-04455-3)
Supplement: Supplementary file 1 — Supplementary Material 1. [file 12883_2025_4455_MOESM1_ESM.docx]

**Additional File 1**. Individual demographic and clinical characteristics of the study participants.

This file provides individual patient data, including demographic and clinical characteristics as well as DBS stimulation parameters set at device activation and at the 6-month follow-up.

| **Subject** | **Sex** | **Age** | **Education** | **Disease duration** | **Side onset** | **UPDRS III Pre off** | **Stimulation parameters at activation STN-LEFT** | **Stimulation parameters at activation STN-RIGHT** | **Stimulation parameters at 6 months STN-LEFT** | **Stimulation parameters at 6 months STN-RIGHT** |
| --- | --- | --- | --- | --- | --- | --- | --- | --- | --- | --- |
|  |  |  |  |  |  |  |  |  |  |  |
|  |  |  |  |  |  |  |  |  |  |  |
| 1 | M | 65-70 | 13 | 15 | R | 43 | C+1- 130/60 1.8 V | C+10- 130/60 1.9 V | C+1 130/60 2.3 V | C+10- 130/60 2.6 V |
| 2 | M | 45-49 | 8 | 12 | L | 41 | C+2- 130/60 1.8 V | C+9- 130/60 1.6V | C+2-130/60 1.95 V | C+ 9- 130/60 1.75 V |
| 3 | M | 60-64 | 18 | 15 | L | 40 | C+2- 130/60 2.2 mA | C+9- 130/60 2 mA | C+2- 130/60 2.2 mA | C+ 9- 130/60 2.0 mA |
| 4 | M | 60-64 | 13 | 10 | L | 18 | C+1- 130/60 2 V | C+9- 130/60 2V | C+1- 130/60 2.1 V | C+ 9- 130/60 2.15 V |
| 5 | M | 60-64 | 8 | 10 | L | // | C+2- 130/60 1.7 V | C+9- 130/60 2.6 V | C+2- 130/60 1.8mA | C+9- 130/60 2.7mA |
| 6 | M | 65-70 | 13 | 16 | R | 41 | C+2- 130/60 1.9 V | C+10-130/60 2.5 V | C+2- 130/60 2.1V | C+10- 130/90 2.8V |
| 7 | F | 65-70 | 13 | 7 | R | 25 | C+2- 130/60 1.9V | C+9- 130/60 1.7 V | C+ 2- 130/60 2.1V | C+ 9- 130/60 1.8V |
| 8 | F | 55-59 | 18 | 10 | R | 40 | C+2- 130/60 2.4 V | C+10-130/60 1.9 V | C+2- 130/60 2.9 V | C+10- 130/60 2.1 V |
| 9 | M | 50-54 | 18 | 12 | L | 45 | C+2- 130/60 2.1 V | C+10-130/60 2.1 V | C+2- 130/60 2.15V | C+10- 130/60 2.15V |
| 10 | M | 55-59 | 8 | 18 | R | // | C+1- 130/60 2.2 V | C+10-130/60 1.8 V | C+1- 130/60 3 V | C+ 10- 130/60 2.5 V |
| 11 | F | 55-59 | 8 | 5 | L | 36 | C+ 2- 130/60 2.1 V | C+11- 130/60 1 V | C+ 2- 130/60 2.3 V | C+11- 130/60 1.3V |
| 12 | F | 50-54 | 18 | 9 | L | 54 | C+1- 130/60 1.3V | C+9- 130/60 1.9V | C+1- 130/60 1.3V | C+ 9- 130/60 1.8V |
| 13 | F | 45-49 | 11 | 8 | L | 48 | C+1- 130/60 1.2 mA | C+9- 130/60 2 mA | C+1- 130/60 1.5mA | C+9- 130/60 2.6mA |
| 14 | F | 60-64 | 13 | 17 | R | 44 | C+0- 130/60 2.3 V | C+10-130/60 2.3 V | C+0- 2.1V 130/60 | C+10- 2.2V 130/60 |
| 15 | M | 55-59 | 18 | 11 | L | // | C+2- 130/60 1.9 V | C+9-130/60 1.7 V | C+2- 130/60 2.3V | C+9- 130/60 1.95V |
| 16 | F | 55-59 | 13 | 11 | R | 39 | C+2- 130/60 1.8 V | C+9-130/60 1.6 V | C+2- 130/60 1.9 V | C+9- 130/60 1.7 V |
| 17 | M | 45-49 | 13 | 11 | R | 19 | C+2- 130 Hz, 60 usec 2.2V | C+9- 130/60 2.2V | C+2- 130 Hz, 60 usec 2.6V | C+9- 130/60 2.5V |
| 18 | M | 45-49 | 13 | 7 | R | 22 | C+1- 130/60 2.1 mA | C+9- 130/60 2mA | C+1- 130/60 2.1 mA | C+9- 130/60 2mA |
| 19 | M | 65-70 | 13 | 19 | L | // | C+2- 130/60 2.1V | C+10- 130/60 2.1V | C+2- 130/60 2.4V | C+10- 130/60 2.4V |
| 20 | M | 45-49 | 18 | 10 | L | // | C+9- 130/60 2 V | C+1- 130/60 2 V | C+9- 130/60 2.6 V | C+1- 130/60 2.8 V |
| 21 | F | 50-54 | 8 | 10 | L | // | C+1- 130/90 2 V | C+10-130/9 1.7 V | C+1- 130/60 2.25V | C+10 130/60 1.95V |
| 22 | M | 65-70 | 13 | 11 | L | 47 | C+2- 130/60 2.5 V | C+ 10- 130/60 2.4 V | C+2- 130/60 2.8 V | C+ 10- 130/60 2.4 V |
| 23 | M | 65-70 | 11 | 10 | L | 55 | C+1- 130/60 1.7 mA | C+ 9- 130/601.8 mA | C+1- 1.7mA | C+ 9- 1.8 mA |
| 24 | F | 70-74 | 11 | 15 | R | 46 | C+3- 130/60 3.4mA | C+10- 130/60 2.3mA | C+3- 130/60 3.3mA | C+10- 130/60 2.3mA |
| 25 | M | 60-64 | 8 | 12 | L | 48 | C+ 1- 130/60 2.2 mA | C+ 10- 130/60 1.7mA | C+ 1- 130/60 2.2 mA | C+ 10- 130/60 1.7mA |

**Notes**. STN= Subthalamic Nucleus; UPDRS= Unified Parkinson’s Disease Rating Scale; M= Male; F= Female; R= Right; L=Left.
